# Supplementary material for: VIVA Stent Preclinical Evaluation in Swine: A Novel Cerebral Venous Stent with a Unique Delivery System
Source: J Clin Med. 2025 Jul 3;14(13):4721. doi: 10.3390/jcm14134721 (PMC12250672; doi:10.3390/jcm14134721)
Supplement: Supplementary file 1 [file jcm-14-04721-s001.zip › jcm-3690047-supplementary.pdf]

Supplementary Table S1 - Thrombogenicity scoring

Score	Thrombus formation description
**0**	Thrombus non-existent or minimal and, if present, appears to be associated with implant venotomy site.
**1**	Thrombus minimal, observed to be covering 1 % to 25 % of material surface.
**2**	Thrombus moderate, observed to be covering 26 % to 50 % of material surface.
**3**	Thrombus severe, observed to be covering 51 % to 75 % of material surface.
**4**	Thrombus extensive, covers 76 % to 100 % of material surface.

Supplementary Table S2 - Semiquantitative histopathological evaluation system - Cell type/response

Cell type / response	Score				
	**0**	**1**	**2**	**3**	**4**
Polymorphonuclear	0	Rare,1–5/phf*	5–10/phf	Heavy infiltrate	Packed
Lymphocytes	0	Rare,1–5/phf*	5–10/phf	Heavy infiltrate	Packed
Plasma Cells	0	Rare,1–5/phf*	5–10/phf	Heavy infiltrate	Packed
Macrophages	0	Rare,1–5/phf*	5–10/phf	Heavy infiltrate	Packed
Giant Cells	0	Rare,1–2/phf	3–5/phf	Heavy infiltrate	Sheets
Necrosis	0	Minimal	Mild	Moderate	Severe

Phf - per high power field

Supplementary Table S3 - Semiquantitative histopathological evaluation system – Tissue response

Cell type / Response	Score				
	**0**	**1**	**2**	**3**	**4**
Adventitial Fibrosis	Absent	Minimal presence of fibrous tissue	Notable fibrous tissue in 25–50% of vessel circumference	Notable fibrous tissue in > 50% of vessel circumference	Not applicable
Fatty Infiltrate	0	Minimal amount of fat associated with fibrosis	Several layers of fat and fibrosis	Elongated and broad accumulation of fat cells at implantation site	Extensive fat completely Surrounding the implantation site

Supplementary Table S4 - Semiquantitative histopathological evaluation system – Additional parameters

Score	**Vascular Injury (0 – 3)[1]**
0	No injury, internal elastic lamina (IEL) intact
1	Disruption of IEL
2	Disruption of tunica media
3	Disruption of the external elastic lamina (EEL) / tunica adventitia
Score[2]	**Endothelialization (endothelial loss) (0-4)**
0	No change was seen
1<br>Minimal	The first (lowest) level of severity in an ordered list based on a five-level scale or up to 25%
2<br>Mild	The second level of severity in an ordered list based on a five-level scale or up to 50%
3<br>Moderate	The third level of severity in an ordered list based on a five-level scale or up to 75%
4<br>Severe	The fourth (highest) level of severity in an ordered list based on a five-level scale or up to 100%
Score	**Stent strut position (1-3)**
1	Superficial in the neointima
2	Middle in the neointima
3	Deep in the neointima

[1] Schwartz RS, Edelman E, Virmani R, et al. Drug-Eluting Stents in Preclinical Studies. Circ Cardiovasc Interv. 2008;1(2):143-153. doi:10.1161/CIRCINTERVENTIONS.108.789974

[2] Schafer KA, Eighmy J, Fikes JD, et al. Use of Severity Grades to Characterize Histopathologic Changes. *Toxicol Pathol*. 2018;46(3):256-265. doi:10.1177/0192623318761348

Score[3]	**Neointimal coverage of the stent strut (i.e., at the circumference of the vessel wall, 0-4)**
0	None
1	up to 25% of the vascular wall thickness
2	up to 50% of the vascular wall thickness
3	up to 75% of the vascular wall thickness
4	up to 100% of the vascular wall thickness
Score	**Luminal Occlusion (1 – 4)**
1	Absent
2	Minimal luminal occlusion (e.g., by fibrin/thrombus and/or neointima) with ~ < 20% compromise lumen area
3	Notable luminal occlusion (e.g., by fibrin/thrombus and/or neointima) with ~ 20% - 50% compromise of lumen area
4	Overwhelming luminal occlusion (e.g., by fibrin/thrombus and/or neointima) with ~ > 50% compromise of lumen area

[3] Liu F, Ge Y, Rong D, et al. Injury and Healing Response of Healthy Peripheral Arterial Tissue to Intravascular Lithotripsy: A Prospective Animal Study. Front Cardiovasc Med. 2022;9. doi:10.3389/fcvm.2022.787973
